# The Effect of Reading Leaflets During the Observation Period After Vaccination on Knowledge of COVID-19 and Vaccines Among Chinese Small Town Residents: A Randomized Controlled Trial

**DOI:** 10.3389/fpubh.2022.819446

**Published:** 2022-03-25

**Authors:** Si-Yi Yu, Jun-Jun Luo, Ke-Shu Shan, Lei Xu, Ling Ding, Xue-Qin Chen

**Affiliations:** ^1^Department of General Internal Medicine, Ningbo First Hospital, Ningbo, China; ^2^Department of Nursing, Ningbo First Hospital, Ningbo, China; ^3^Department of Traditional Medicine, Ningbo First Hospital, Ningbo, China

**Keywords:** COVID-19, COVID-19 vaccine, leaflet, health education, randomized controlled trial (RCT)

## Abstract

**Background:**

Public health education is essential for epidemic prevention and control in the post-COVID-19 era. This randomized controlled trial (RCT) aims to evaluate the effect of reading leaflets during the observation period after vaccination on knowledge of COVID-19 disease and vaccines in Chinese small town residents and to provide suggestions for public health education.

**Methods:**

Residents were recruited during the observation period after getting vaccinated against COVID-19 in Xidian and were randomly divided into an education group and a control group. The education group was asked to complete the questionnaire after reading a leaflet, whereas the control group directly completed the questionnaire. The questionnaire comprised two sections on COVID-19 knowledge and vaccine knowledge, and the scores were used to assess the resident's knowledge.

**Results:**

A total of 142 participants in the education group and 154 participants in the control group were enrolled. The rates of correct knowledge in the education group and the control group were 90.7 and 83.1%, respectively. The scores of the two sections and the aggregate knowledge score of the education group were significantly higher than those of the control group (*P* < 0.001). The rates of correct responses to questions on clinical manifestations and transmission routes of COVID-19 and indications and contraindications of vaccines significantly increased after reading the leaflets (*P* < 0.05). Knowledge of different groups of genders, ages, marital statuses, education levels and occupations all improved (*P* < 0.05), and the 18–29-year-old and never-married group revealed a higher growth rate of correct responses.

**Conclusion:**

Chinese small town residents have a median level of knowledge regarding COVID-19 disease and vaccines. Reading leaflets during the observation period after vaccination effectively improved their knowledge. This low-cost and efficient health education approach can be widely applied.

## Introduction

Coronavirus disease 2019 (COVID-19) is a respiratory disease caused by a novel coronavirus named severe acute respiratory syndrome coronavirus 2 (SARS-CoV-2). The virus spreads rapidly through respiratory droplets and close contact with diversified transmission routes and strong interpersonal infectivity (1). By October 28, 2021, COVID-19 had resulted in 245,373,039 infections and 4,979,421 deaths worldwide (2).

To relieve the burden of COVID-19 and the depletion of medical resources, a total of 6,838,727,352 vaccine doses were administered worldwide by October 28, 2021 (2). China has extensively promoted COVID-19 vaccination, which has been free for all people since January 2021 (3). A total of 2,257,584,000 doses of COVID-19 vaccines were administered in China by October 28, 2021 (4). China has generally controlled the COVID-19 epidemic due to widespread vaccination and other containment strategies despite localized outbreaks (5). Nonetheless, people's knowledge of COVID-19 disease and vaccines may affect their adherence to public health measures (6). Therefore, effective health education remains essential in the post-COVID-19 era (7, 8).

Currently, studies have focused on the impact of online health education on people's attitudes and practices toward the COVID-19 epidemic. A study from Iran showed that social media such as Instagram played a crucial role in public health education for COVID-19 (9). A Chinese online study revealed that watching health education videos helped establish a healthier mental state and health-related behaviors against COVID-19 including mask-wearing, disinfection, temperature-taking, etc. (10). Another ongoing randomized controlled trial (RCT) focused on the effect of animated videos on good COVID-19 hygiene habits (11). However, these studies were conducted online due to the raging epidemic, and real and effective offline COVID-19 education remains lacking. People who get vaccinated need to be monitored for at least 15 to 30 min in case of possible serious allergic reactions (12, 13), and the observation period provides an appropriate opportunity for COVID-19 education.

Xidian town, Zhejiang Province is a small town dominated by an industrial economy on the southeast coast of China. The total population is approximately 100,000, including 55,000 immigrants (mainly migrant workers) with a relatively low education level (14). We conducted an RCT among residents who were vaccinated against COVID-19 in Xidian to determine the effect of reading leaflets during the observation period after vaccination on the knowledge of COVID-19 disease and vaccines. We expected to provide suggestions for public health education in the post-COVID-19 era.

## Methods

### Participants

This RCT was conducted at the Xidian vaccination site between August 18, 2021 and September 8, 2021. Residents vaccinated against COVID-19 were recruited. Individuals who were below 18 years of age, unable to read or understand the study, or unwilling to participate were excluded. The study complied with the Declaration of Helsinki and was approved by the Ethics Committee of Ningbo First Hospital. All participants signed an informed consent form before the survey. This study was registered on ClinicalTrials.gov (NCT05033860) and conformed to the CONSORT statement (15).

### Measures

The participants were randomly assigned to the education group (intervention group) or control group at a 1:1 ratio. The grouping scheme was generated from random computer-generated sequences and placed into light-tight sealed envelopes by a researcher who was not involved in the field investigation. All the investigators received unified training. Subjects in the education group were asked to complete a questionnaire (Table 1) after reading a leaflet (Supplementary Figure 1), whereas those in the control group directly completed the questionnaire. There was no time limit for reading the leaflet or completing the questionnaire. Participants could ask the researchers for help if they had questions about the leaflet.

**Table 1:** Questionnaire of knowledge toward COVID-19 disease and vaccines.

**Questions**	**Options**
K1.1 The main clinical symptoms of COVID-19 are fever, fatigue, dry cough, and myalgia.	True, false, I don't know
K1.2 Unlike the common cold, stuffy nose, runny nose, and sneezing are less common in persons infected with the COVID-19 virus.	True, false, I don't know
K1.3 There is currently no effective cure for COVID-19, but early symptomatic and supportive treatment can help most patients recover from the infection.	True, false, I don't know
K1.4 Not all persons with COVID-19 will develop to severe cases. Elderly people, patients with chronic diseases, and obese people are more likely to be severe cases.	True, false, I don't know
K1.5 It is uncertain whether wild animals are the source of COVID-19 infection.	True, false, I don't know
K1.6 Persons with COVID-19 cannot transmit the virus to others when a fever is not present.	True, false, I don't know
K1.7 The COVID-19 virus spreads via respiratory droplets of infected individuals.	True, false, I don't know
K1.8 Residents can wear general medical masks to prevent the infection by the COVID-19 virus.	True, false, I don't know
K1.9 It is not necessary for children and young adults to take measures to prevent the infection by the COVID-19 virus.	True, false, I don't know
K1.10 To prevent the infection by COVID-19, individuals should avoid going to crowded places such as train stations and avoid taking public transportations.	True, false, I don't know
K1.11 Isolation and treatment of people who are infected with the COVID-19 virus are effective ways to reduce the spread of the virus.	True, false, I don't know
K1.12 People who have contact with someone infected with the COVID-19 virus should be immediately isolated in a proper place. In general, the observation period is 14 days.	True, false, I don't know
K2.1 The COVID-19 vaccination is legally mandatory.	True, false, I don't know
K2.2 The COVID-19 vaccination is indicated in infants below 1 year of age.	True, false, I don't know
K2.3 The COVID-19 vaccination is indicated in women who are preparing for pregnancy or lactating mothers.	True, false, I don't know
K2.4 The COVID-19 vaccination is indicated in patients with acute infection.	True, false, I don't know
K2.5 The COVID-19 vaccination is indicated in patients with chronic diseases, such as diabetes, hypertension and heart diseases.	True, false, I don't know
K2.6 The COVID-19 vaccination is indicated in person who has already recovered from COVID-19.	True, false, I don't know
K2.7 The COVID-19 vaccination is indicated in immunocompromised patients.	True, false, I don't know
K2.8 The COVID-19 vaccination is indicated in person allergic to vaccine components.	True, false, I don't know
K2.9 For COVID-19 vaccines that require two injections, a better immune effect can be obtained after two injections.	True, false, I don't know
K2.10 Mild side effects, such as arm pain, redness, fatigue, headache, muscle pain, chills, fever, and nausea, may occur after COVID-19 vaccination.	True, false, I don't know
K2.11 Other measures (such as wearing a mask and avoiding crowded places) are still important after COVID-19 vaccination.	True, false, I don't know
K2.12 Currently the COVID-19 vaccines are effective in preventing the disease.	True, false, I don't know

The leaflet comprised two sections: knowledge of COVID-19 and knowledge of COVID-19 vaccines. COVID-19 knowledge included the clinical manifestations, transmission routes, and prevention and control strategies of COVID-19. It was based on a previous study (9) and was excerpted from *the Guidelines for the Diagnosis and Treatment of Coronavirus Disease 2019 (trial version eighth)* and the revised version published by the National Health Commission of the People's Republic of China (PRC) (16, 17). Vaccine knowledge included China's COVID-19 vaccination policy, indications and contraindications, adverse reactions, effects, and postvaccination prevention and control measures. It was drawn from *Guidelines of Vaccination for COVID-19 Vaccines in China (First edition)* published by the National Health Commission of the PRC (18).

The questionnaire consisted of two sections: demographics and knowledge. Demographic variables included age, gender, marital status, education, occupation, and medical history. The knowledge assessment included two sections: knowledge of COVID-19 and knowledge of COVID-19 vaccines (Table 1). COVID-19 knowledge was excerpted from a previous study (19), and had 12 questions: 4 regarding clinical manifestations (K1.1-K1.4), 3 regarding transmission routes (K1.5-K1.7), and 5 regarding the prevention and control of COVID-19 (K1.8-K1.12). Vaccine knowledge also had 12 questions: 1 regarding COVID-19 vaccination policy in China (K2.1), 7 regarding indications and contraindications for the vaccine (K2.2-K2.8) (based on a previous study (20)), 1 regarding vaccine adverse reactions (K2.10), 2 regarding vaccine effectiveness (K2.9, K2.12), and 1 regarding postvaccination prevention and control measures (K2.11). The options for answers included “True/False/I don't know.” A correct answer was scored 1 point, whereas an incorrect/unknown answer was scored 0 points. The total score of each section was between 0 and 12 points, and a higher score represented better knowledge. The Cronbach's alpha coefficient of the knowledge section was 0.756 in our unpublished research with 405 participants, indicating acceptable internal consistency (21).

The sample size was based on a preliminary experiment with 42 participants. PASS software (α = 0.05, 1-β = 0.9) was used, and the sample size was calculated according to the knowledge scores of the two sections (effective size for COVID-19 part and vaccine part were 0.54 and 1.63, respectively). Selecting the larger number as the sample size, each group had 126 participants. Accordingly, the number of participants in each group was 158 with an assumed loss to follow-up rate of 20%, and the total sample size was 316.

### Statistical Analysis

Data were analyzed by IBM SPSS (version 26.0). Continuous variables are presented as the mean ± standard deviation (SD). Independent sample *T* tests, one-way analysis of variance (ANOVA) or chi-square tests were adopted to compare the knowledge scores of different groups where appropriate. When comparing the increase in scores, a conditional pair comparison was performed between the education group and the control group in advance. *P* < 0.05 was considered statistically significant.

## Results

**Figure 1:**
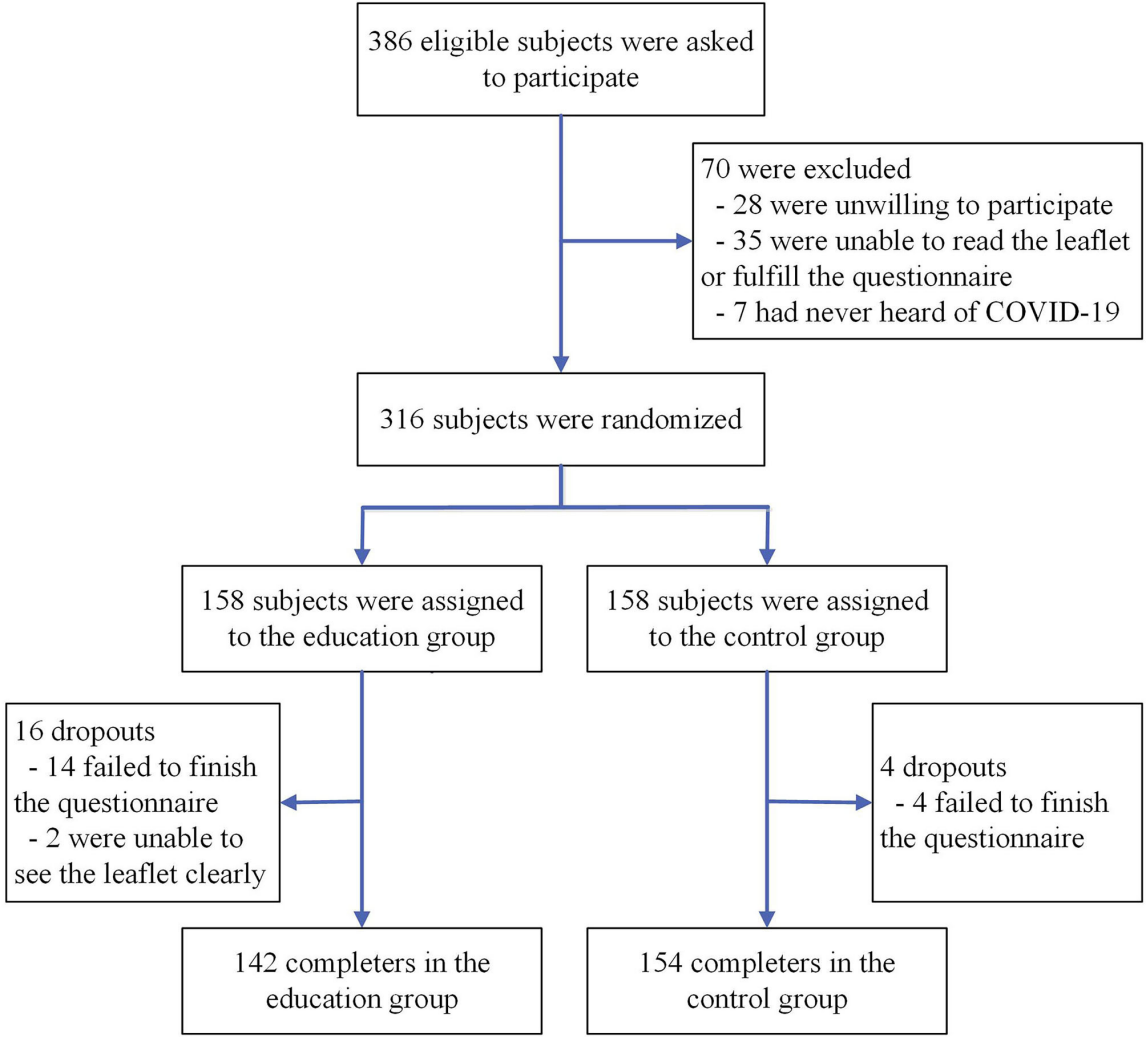
Inclusion flow chart.

The inclusion flow chart is shown in Figure 1. A total of 386 eligible residents were asked to participate in the research, 70 of whom were excluded according to the exclusion criteria (28 were unwilling to participate, 35 were unable to read or complete the questionnaire, and 7 had never heard of COVID-19). Among the 316 participants included, 158 were randomly assigned to the education group and the control group, separately. Excluding 14 subjects who suspended the survey in the education group, 2 subjects who could not see the leaflet clearly and 4 subjects who suspended the survey in the control group, 142 subjects in the education group and 154 subjects in the control group eventually completed the study. The loss to follow-up rates of the two groups were 10.1 and 2.5%, respectively. Among the 296 responders, the average age was 37.4 years (SD: 11.9, range: 18–83). In total, 165 respondents (55.7%) were male, 181 (61.1%) possessed a degree of middle school or below, 164 (55.4%) engaged in physical labor, and 27 (9.1%) had chronic diseases. No significant differences in the demographic characteristics were revealed between the two groups (Table 2, *P* > 0.05).

**Table 2:** Baseline characteristics of residents included in this study.

	**Total study population**	**Control group**	**Education group**	***P*** **-value**
	***n*** **= 296**	***n*** **= 154**	***n*** **= 142**	
**Male**, ***n*** **(%)**	165 (55.7%)	83 (53.9%)	82 (57.7%)	0.505
**Age**, ***n*** **(%)**				
18–29 years	86 (29.1%)	46 (29.9%)	40 (28.2%)	0.946
30–49 years	157 (53%)	81 (52.6%)	76 (53.5%)	
50+ years	53 (17.9%)	27 (17.5%)	26 (18.3%)	
**Marital status**, ***n*** **(%)**				
Married	226 (76.4%)	119 (77.3%)	107 (75.4%)	0.545
Never-married	66 (22.3%)	32 (20.8%)	34 (23.9%)	
Divorced or widowed widowed	4 (1.4%)	3 (1.9%)	1 (0.7%)	
**Education**, ***n*** **(%)**				
Primary school and below	51 (17.2%)	27 (17.5%)	24 (16.9%)	0.877
Middle school	130 (43.9%)	66 (42.9%)	64 (45.1%)	
High school	77 (26%)	39 (25.3%)	38 (26.8%)	
Bachelor' degree and above	38 (12.9%)	22 (14.3%)	16 (11.3%)	
**Occupation**, ***n*** **(%)**				
Physical labor	164 (55.4%)	81 (52.6%)	83 (58.5%)	0.516
Mental labor	76 (25.7%)	45 (29.2%)	31 (21.8%)	
Students	7 (2.4%)	3 (1.9%)	4 (2.8%)	
Unemployed	49 (16.6%)	25 (16.2%)	24 (16.9%)	
**Medical history**, ***n*** **(%)**	27 (9.1%)	13 (8.4%)	14 (9.9%)	0.672

**Figure 2:**
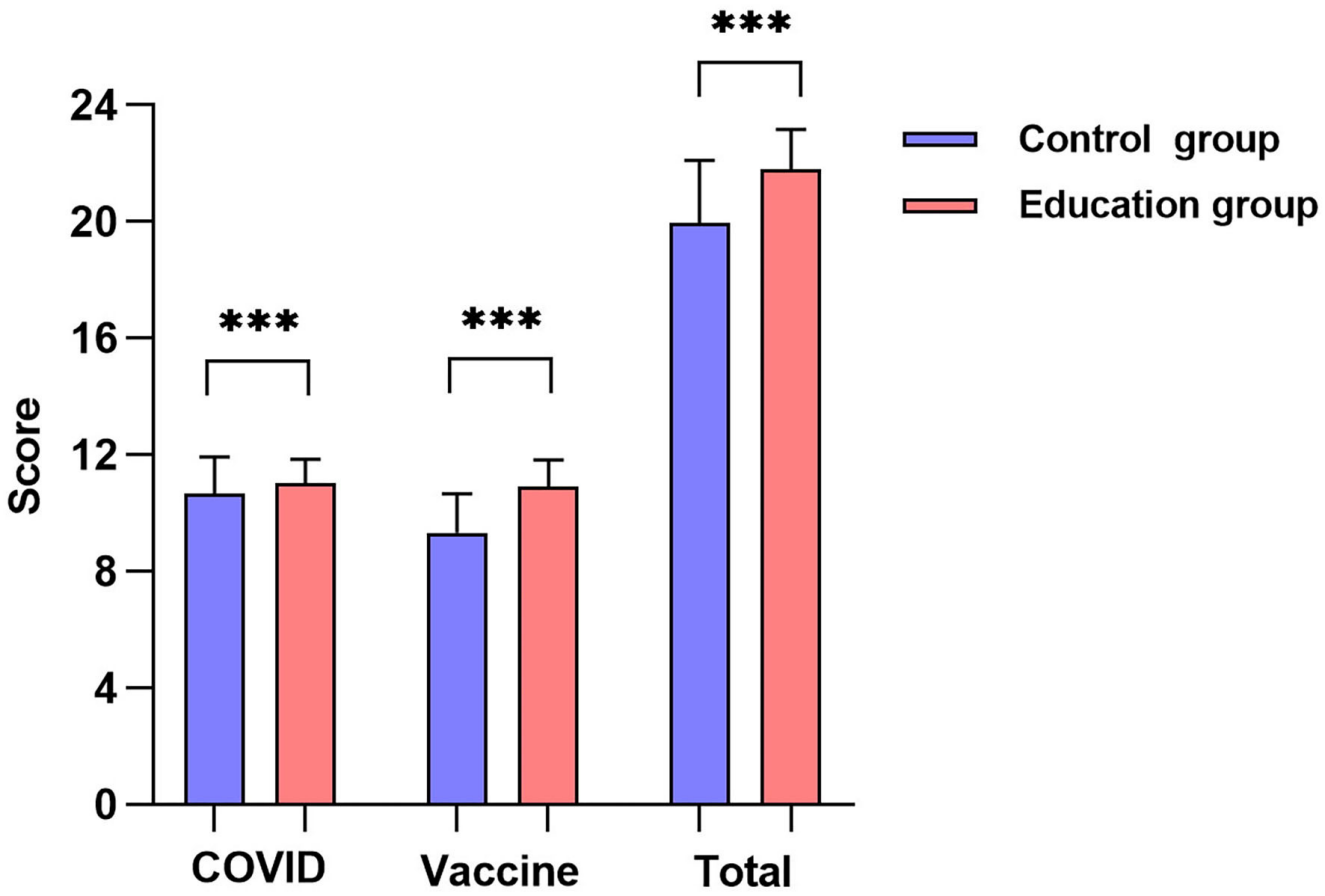
Knowledge score of education group and control group (***P < 0.001).

The average aggregate knowledge scores of the control group and the education group were 19.94 (SD: 2.19, range: 12–24) and 21.77 (SD: 1.42, range: 17–24), respectively. The overall correct rates were 83.1 and 90.7%, respectively. The average scores of the 12 questions about COVID-19 knowledge were 10.49 (SD: 1.29, range: 5–12) and 10.98 (SD: 0.87, range: 8–12), respectively, and those about vaccine knowledge were 9.25 (SD: 1.41, range: 4–12) and 10.79 (SD: 0.98, range: 8–12), respectively. The scores of the two sections and the aggregate score of the education group were significantly higher than those of the control group (Figure 2, *P* < 0.001).

**Figure 3:**
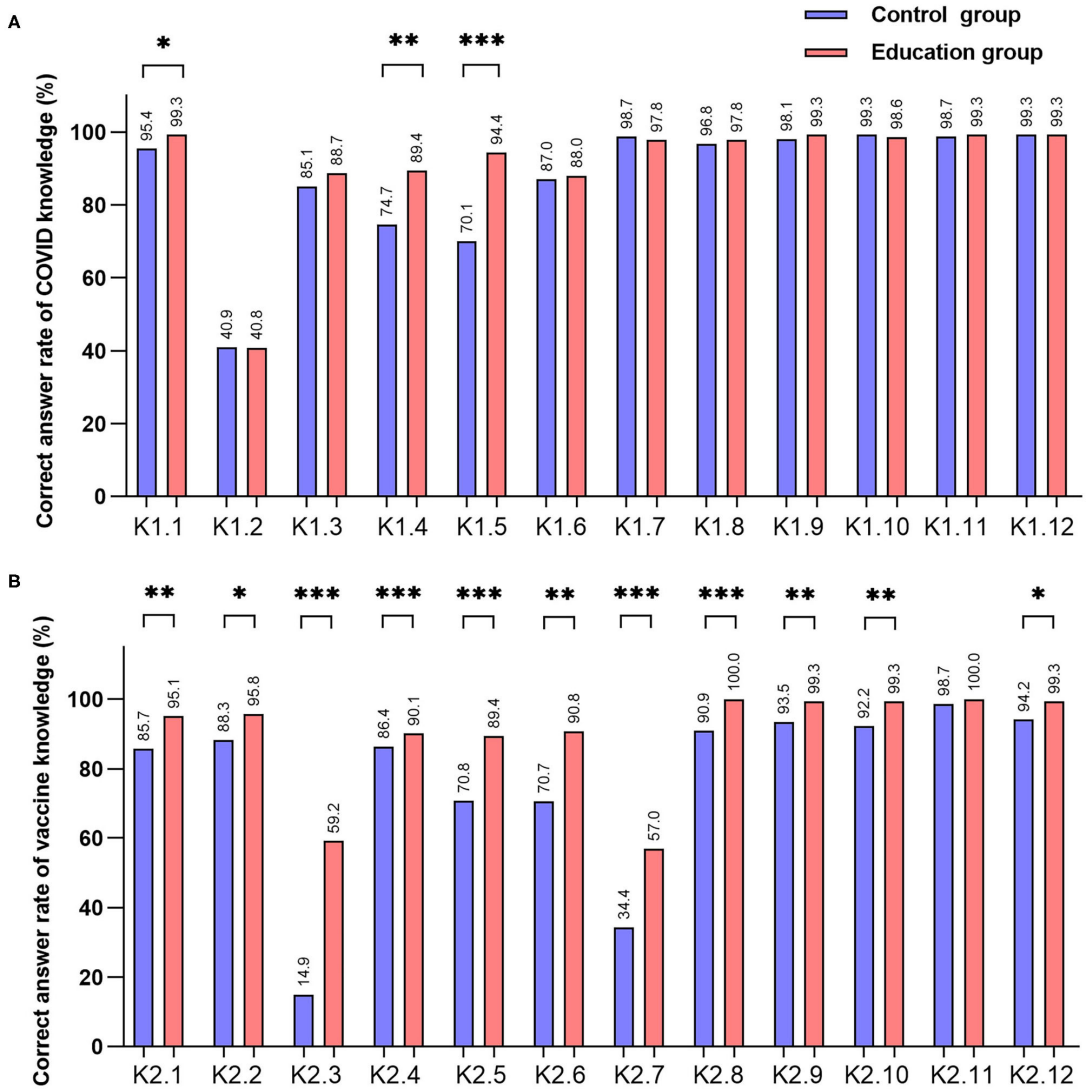
Correct rate of knowledge by each question. [*P < 0.05, **P < 0.01, ***P < 0.001 **(A)** correct answer rates of 12 questions of COVID-19 knowledge, **(B)** correct answer rates of 12 questions of vaccine knowledge].

Regarding COVID-19 knowledge, the rates of correct responses to K1.2, K1.4, and K1.5 about clinical manifestations and transmission routes were low, whereas correct response rates to questions about epidemic prevention and control were relatively high. The correct response rates to K1.1, K1.4, and K1.5 significantly increased after reading the leaflet (Figure 3A, *P* < 0.05), whereas no significant change was revealed in other questions (Figure 3A, *P* > 0.05). For vaccine knowledge, the correct response rates to K2.3, K2.5, K2.6, and K2.7 about indications and contraindications were low, whereas correct response rates to questions about vaccination policies, effects, adverse reactions, and postvaccination prevention and control measures were relatively high. The correct response rates to questions in the vaccine section generally increased after education (Figure 3B, *P* < 0.05), except for K2.11.

The COVID-19 knowledge scores and aggregate scores differed significantly across ages and marital status (Supplementary Table 1, *P* < 0.05) but not across genders, education levels, occupations or disease statuses. Due to the limited number of divorced or widowed participants, we eliminated these respondents and then performed multiple linear regression analyses (Supplementary Table 2). The age group of 18–29 years (vs. the age group of 30–49 years, β:−0.260, *P* < 0.01) was associated with a lower COVID-19 knowledge score, and the never-married group was correlated with a lower vaccine knowledge score (vs. married group, β:−0.254, *P* < 0.01) and a lower aggregate knowledge score (vs. married group, β:−0.302, *P* < 0.001). Besides, the education of high school was correlated with a lower vaccine knowledge score (vs. bachelor's degree, β:−0.608, *P* < 0.01).

**Figure 4:**
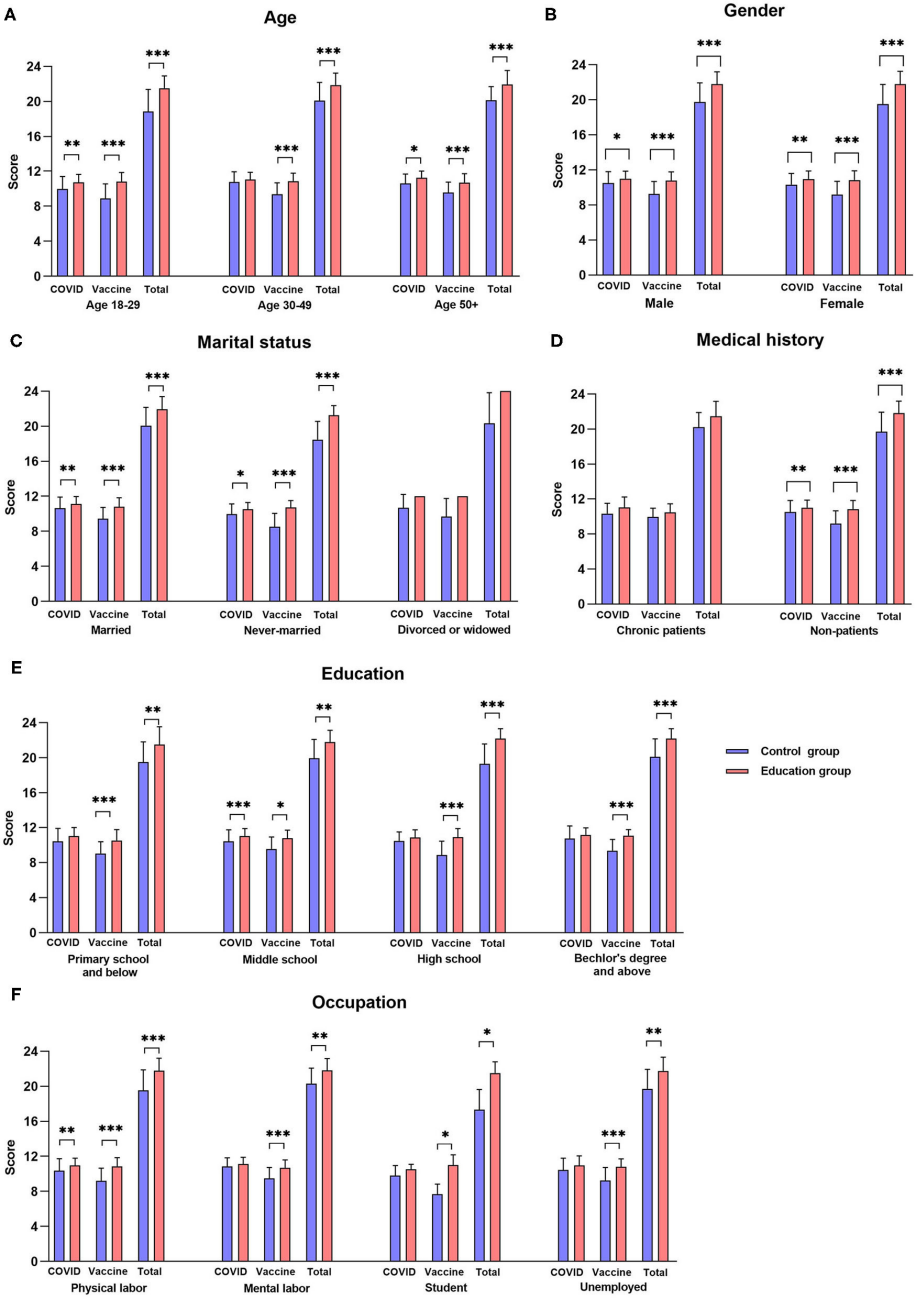
Knowledge score of education group and control group by demographic variables. [*P < 0.05, **P < 0.01, ***P < 0.001, **(A)** scores of different ages, **(B)** scores of different genders, **(C)** scores of different marital statuses, **(D)** scores of different medical histories, **(E)** scores of different education levels, **(F)** scores of different occupations].

The COVID-19 knowledge score of the age group of 30–49 years failed to show an increase (Figure 4A, *P* > 0.05), whereas that of the younger and older populations increased significantly after education (Figure 4A, *P* < 0.05). The vaccine knowledge scores and aggregate knowledge scores of different age groups generally increased after education (*P* < 0.001), but with different increase rates (Table 3, *P* < 0.05). The increase in the vaccine knowledge score of age group of 18–29 years was significantly greater than the age group of 50+ years, and that of the aggregate knowledge score was significantly greater than age group of 30–49 years and 50+ years (*P* < 0.05). The scores of the two sections and aggregate knowledge of males and females increased significantly after reading the leaflet (Figure 4B, *P* < 0.05) with no significant difference in the rate of increase (Table 3, *P* > 0.05). The knowledge scores of both married and never-married persons increased significantly after education (Figure 4C, *P* < 0.01), whereas those of divorced or widowed persons did not change significantly (*P* > 0.05). The increases in the vaccine knowledge score and aggregate knowledge score of never-married people were significantly greater than those of married people (Table 3, *P* < 0.05). Unlike chronic patients, non-patients showed significantly higher knowledge scores after education (Figure 4D, *P* < 0.01). For people with different education levels, the COVID-19 knowledge score of those with a middle school degree significantly increased after education (Figure 4E, *P* < 0.001), whereas those with other educational backgrounds did not change (*P* > 0.05). The vaccine knowledge scores and the aggregate knowledge scores of people with different education levels generally increased after education (*P* < 0.05). For subjects with different occupations, the COVID-19 knowledge score of physical workers increased significantly after education (Figure 4F, *P* < 0.01), whereas those of other occupations did not change (*P* > 0.05). The vaccine knowledge scores and the aggregate knowledge scores of different occupational groups drastically increased after education (*P* < 0.05). The increase in knowledge scores did not reveal any differences across education levels and occupations (Table 3, *P* > 0.05).

**Table 3:** Increase of knowledge score by demographic variables.

**Demographic variables**		**Increase of COVID score**	**Increase of vaccine score**	**Increase of aggregate score**
		**(mean ±SD)**	**(mean ±SD)**	**(mean ±SD)**
Gender	Male	0.39 ± 1.27	1.43 ± 1.65	1.82 ± 2.33
	Female	0.61 ± 1.41	1.70 ± 1.84	2.31 ± 2.53
Age (years)	18–29	0.89 ± 1.62	2.09 ± 1.79^*^	2.97 ± 2.53^*^
	30–49	0.29 ± 1.35	1.53 ± 1.53	1.82 ± 2.32
	50+	0.71 ± 1.46	0.95 ± 1.27	1.67 ± 2.16
Marital status	Married	0.43 ± 0.69	1.30 ± 1.71^*^	1.74 ± 2.61^*^
	Never-married	0.69 ± 1.49	2.17 ± 1.75	2.86 ± 2.40
Education	Primary school and below	0.70 ± 2.01	1.52 ± 1.88	2.22 ± 3.34
	Middle school	0.69 ± 1.46	1.19 ± 1.60	1.87 ± 2.36
	High school	0.39 ± 1.25	2.12 ± 1.82	2.52 ± 2.53
	Bachelor' degree and above	0.09 ± 1.14	1.73 ± 1.01	1.82 ± 1.66
Occupation	Physical labor	0.50 ± 1.60	1.50 ± 1.62	2.00 ± 2.50
	Mental labor	0.48 ± 1.21	1.21 ± 1.63	1.69 ± 2.22
	Students	1.00 ± 1.00	3.00 ± 0.00	4.00 ± 1.00
	Unemployed	0.57 ± 1.54	1.57 ± 1.72	2.14 ± 2.71

* *P < 0.05, SD, standard deviation*.

## Discussions

To the best of our knowledge, this is the first RCT on COVID-19 education in the observation period after vaccination. In this population with relatively low education and occupations predominantly involving physical labor, residents lacked some knowledge about COVID-19 disease and vaccines. After reading the leaflet, the resident's knowledge of COVID-19 disease and vaccines both improved significantly.

In our study, small town residents had relatively poor knowledge of the clinical manifestations and transmission routes of COVID-19 and good knowledge of prevention and control measures. Due to the strict prevention measures by the local government and the lack of COVID-19 outbreaks in Zhejiang Province for more than a year, most people knew a considerable amount of information about epidemic prevention and control measures but were unfamiliar with the symptoms and transmission routes of the disease. This finding indicated the inadequacy of COVID-19 education for small town residents (22). After reading the leaflet, people's knowledge about COVID-19 improved significantly. Residents lacked knowledge of vaccine indications and contraindications, but had a good grasp of vaccination policies, vaccine effects, adverse reactions, and postvaccination prevention and control measures. The reason might be that people gained knowledge about COVID-19 vaccines due to public health education and widespread vaccination, but still relied on medical professionals for guidance on vaccine indications and contraindications. After reading the leaflet, resident's knowledge about vaccines generally improved.

The knowledge of residents of different genders, ages, marital statuses, education levels and occupations improved dramatically after reading the leaflet, suggesting the applicability of the health education approach in all population groups. The knowledge level of chronic patients and divorced or widowed people did not show significant increases after education, which might be attributed to the relatively small sample size. The increase in knowledge was greater in young individuals compared with elderly individuals likely because adolescents have a better capability of accepting new knowledge. In addition, the elderly had better health knowledge before education, which resulted in a relatively minimal increase in knowledge after education. Never-married people gained more knowledge than married people after education, which might be correlated with their age.

The health education approach we proposed has the following strengths. First, our findings show that by reading the leaflet during the observation period after vaccination, resident's knowledge of COVID-19 disease and vaccines effectively improved. The opportunity for offline health education is lacking due to the impact of the COVID-19 epidemic, in which case the observation period after vaccination provides an appropriate occasion for education. Our offline education approach is more effective and convenient than online COVID-19 education. Resident's questions about health knowledge can be quickly and effectively answered by medical professionals, which also improves the efficiency of public health workers. Second, this is a low-cost health education approach. Residents can read the leaflet within a 30-min observation period after vaccination without extra time for learning, which is more acceptable. Third, this health education method is especially suitable for small town residents with low education levels. Comprising a considerable proportion of the Chinese population, small town residents lack knowledge about COVID-19 disease and vaccines due to their limited acceptance of health knowledge from the media and reduced access to online health resources (23). The paper leaflet is simple and easy to understand, providing a proper channel to gain knowledge of COVID-19 prevention.

One limitation of our research is that we only conducted the study in Xidian instead of multiple centers. Several groups, including divorced or widowed people, students, and chronic patients, had a relatively small sample size, leading to unreliable knowledge evaluation data. Another limitation is that some statements in our questionnaire, such as K2.12, was more likely to measure perception than knowledge. This would lead to biases in the assessment of knowledge.

## Conclusion

Chinese small town residents lacked some knowledge of COVID-19 disease and vaccines, and reading leaflets during the observation period after vaccination effectively improved the knowledge among different groups of genders, ages, marital status, education level, and occupations. This effective and low-cost health education approach can be applied to improve public knowledge of COVID-19 disease and vaccines. Multicenter and stratified studies involving different populations are required to confirm the effectiveness of this approach.

## Data Availability Statement

The raw data supporting the conclusions of this article will be made available by the authors, without undue reservation.

## Ethics Statement

The studies involving human participants were reviewed and approved by the Ethics Committee of Ningbo First Hospital. The patients/participants provided their written informed consent to participate in this study.

## Author Contributions

S-YY designed the survey and drafted the manuscript. S-YY, J-JL, and LD distributed the questionnaires and organized the data. S-YY and K-SS performed the statistical analyses. LX and X-QC supervised the study design and conduction. All authors contributed to the article and approved the submitted version.

## Funding

This work was supported by the Zhejiang Medical Health Science and Technology Program (No. 2021KY992) and Ningbo Natural Science Foundation of China (Nos. 2019A610221 and 2019Y32).

## Conflict of Interest

The authors declare that the research was conducted in the absence of any commercial or financial relationships that could be construed as a potential conflict of interest.

## Publisher's Note

All claims expressed in this article are solely those of the authors and do not necessarily represent those of their affiliated organizations, or those of the publisher, the editors and the reviewers. Any product that may be evaluated in this article, or claim that may be made by its manufacturer, is not guaranteed or endorsed by the publisher.
