# Supplementary material for: The Effect of Reading Leaflets During the Observation Period After Vaccination on Knowledge of COVID-19 and Vaccines Among Chinese Small Town Residents: A Randomized Controlled Trial
Source: Front Public Health. 2022 Mar 25;10:819446. doi: 10.3389/fpubh.2022.819446 (PMC8990908; doi:10.3389/fpubh.2022.819446)
Supplement: Supplementary file 1 [file Data_Sheet_1.pdf]

Supplementary Material

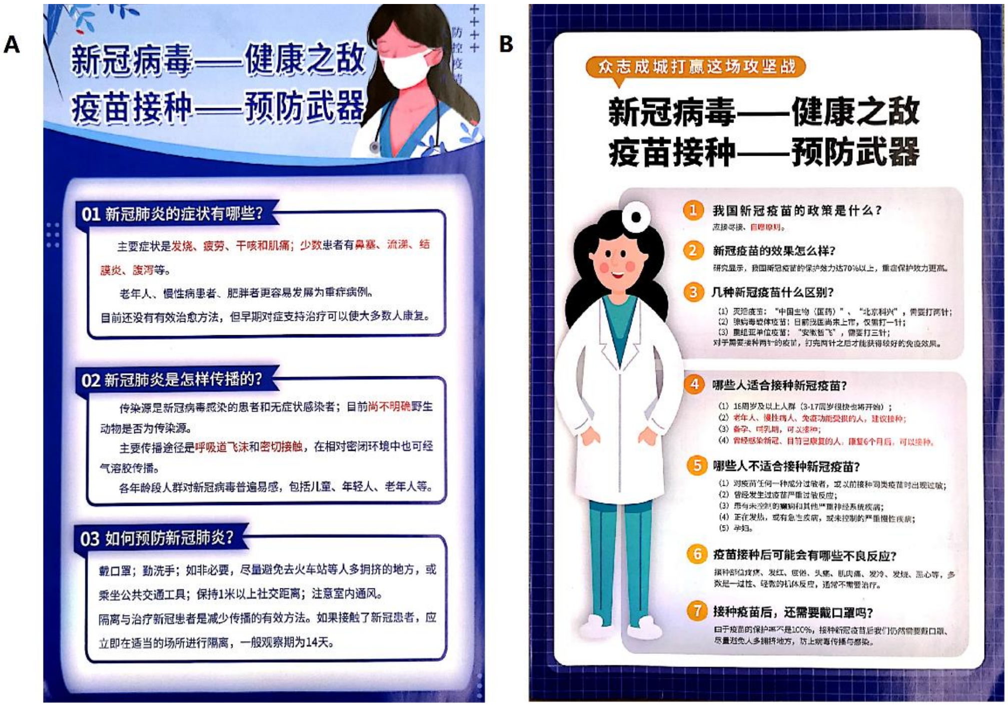

Supplementary Figure 1 Informational leaflets with knowledge of COVID-19 and vaccines

(A: Front page of the leaflet, B: Back page of the leaflet)

Supplementary Figure 1 (in English)

Front page: COVID-19 as Enemy of Health, Vaccination as Preventive Weapon

1. What are the symptoms of COVID-19?

The main symptoms are fever, fatigue, dry cough and myalgia; a few patients may have nasal stuffy or runny nose, conjunctivitis, diarrhea, etc. Elderly people, patients with chronic diseases, and obese people are more likely to be severe cases. There is currently no effective cure for COVID-19, but early symptomatic and supportive treatment can help most patients recover from the infection.

2. How is the COVID-19 virus spread?

The infection sources include patients and asymptomatic carriers. It is not yet clear whether wild animals are the source of infection. The main transmission routes are respiratory droplets and close contact as well as aerosols in a relatively closed environment. People of all ages are susceptible to COVID-19.

3. How to prevent COVID-19?

Wearing a mask; washing hands frequently; avoiding going to crowded places or taking public transportation; keeping a social distance of more than one meter; ventilating frequently. Isolation and treatment of infected people are effective methods to reduce the spread of the virus. Those who have contact with infected persons should be immediately isolated in a proper place, and the observation period is generally 14 days.

Back page:

1. What is China's policy on COVID-19?

People who require vaccination should all be vaccinated, and vaccination should be voluntary.

2. What is the effect of the COVID-19 vaccine?

Studies have shown that the protective efficacy of China's COVID-19 vaccine is up to 70%, and the protective efficacy of is even higher for high-risk cases.

3. What is the difference between various COVID-19 vaccines?

(1) Inactivated vaccines are manufactured by "China Biotech" and "Sinovac Biotech" and require two injections.

(2) Adenovirus vaccines require one injection and are not yet listed in China.

(3) Recombinant subunit vaccines are manufactured by "Anhui Zhifei Longcom" and require three injections.

For vaccines that require two injections, a better immune effect can be obtained after two injections.

4. Who is eligible for the COVID-19 vaccine?

(1) People aged 18 and above (indications for 3-17 years will be approved soon);

(2) Elderly, chronic patients, and immunocompromised people are recommended to be vaccinated;

(3) Women who are preparing for pregnancy or lactating mothers can be vaccinated;

(4) People who have already recovered from COVID-19 for longer than 6 months can be vaccinated.

5. Who is contraindicated for the COVID-19 vaccine?

(1) People who are allergic to any component of the vaccine, or experienced anaphylaxis after vaccination with similar vaccines;

(2) People who have experienced severe anaphylaxis to vaccines;

(3) Patients who suffer from uncontrolled epilepsy and other serious neurological diseases;

(4) Patients who have fever, acute illness, or uncontrolled serious chronic illness;

(5) Pregnant women.

6. What are the possible adverse reactions after vaccination?

Mild side effects, such as arm pain, redness, fatigue, headache, muscle pain, chills, fever, and nausea, may occur after COVID-19 vaccination. These symptoms typically disappear without treatment in several days.

7. Do I still need to wear a mask after vaccination?

Because the protective rate of the COVID-19 vaccine is not 100%, we still need to wear masks after vaccination. We should also avoid crowded places to prevent infection.

Supplementary Table 1 Knowledge score of COVID-19 and vaccines of control group by demographic variables

Demographic variables		Knowledge score of COVID (mean ± SD)	Knowledge score of vaccines (mean ± SD)	Aggregate knowledge score (mean ± SD)
**Gender**	Male	10.58±1.27	9.31±1.33	19.89±2.12
	Female	10.28±1.28	9.08±1.57	19.37±2.33
**Age (years)**	18-29	9.93±1.42 **	8.80±1.73	18.74±2.58 **
	30-49	10.73±1.17	9.32±1.29	20.05±2.06
	50+	10.44±1.09	9.56±1.19	20.00±1.59
**Marital status**	Married	10.59±1.29 *	9.39±1.33 **	19.98±2.13 **
	Never-married	9.88±1.07	8.47±1.59	18.34±2.04
	Divorced or widowed	10.67±1.53	9.67±2.08	20.33±3.51
**Education**	Primary school and below	10.30±1.46	9.00±1.33 *	19.30±2.30
	Middle school	10.39±1.31	9.52±1.36	19.91±2.18
	High school	10.44±1.02	8.74±1.65	19.18±2.35
	Bachelor' degree	10.77±1.38	9.36±1.22	20.14±1.96
**Occupation**	Physical labor	10.30±1.37	9.16±1.50	19.46±2.34 *
	Mental labor	10.78±1.00	9.44±1.24	20.22±1.76
	Students	9.67±1.16	7.33±1.53	17.00±2.65
	Unemployed	10.40±1.35	9.16±1.49	19.56±2.33
**Medical history**	Yes	10.07±1.21	9.71±1.38	19.79±1.93
	No	10.48±1.28	9.16±1.44	19.64±2.26

*P<0.05, **P<0.01, SD: standard deviation

Supplementary Table 2 Multiple linear regression on factors associated with knowledge of COVID-19 and vaccines

Variable	Coefficient	Standard error	β	t	P
**Knowledge score of COVID**					
Age group (18-29 vs. 30-49 years)	-0.723	0.218	-0.260	-3.314	0.001
**Knowledge score of vaccine**					
Marital status (never-married vs. married)	-0.899	0.278	-0.254	-3.228	0.002
Education (primary school and below vs. bachelor's degree and above)	-0.584	0.303	-0.155	-1.926	0.056
Education (high school vs. bachelor's degree and above)	-0.608	0.267	-0.184	-2.280	0.024
**Aggregate knowledge score**					
Marital status (never-married vs. married)	-1.648	0.423	-0.302	-3.900	<0.001
